# 

**Published:** 1876-08

**Authors:** 


*CONTENTS.*

| | Page |
|---|---|
| The Poor Babies . . . . | 331 |
| Weak Lungs . . . . . . . | 333 |
| A Remedy for Every Ill | 334 |
| Unhealthy Localities . . | 335 |
| Profanity . . . . . . . . | 337 |
| Early Rising . . . . . . | 337 |
| Drunken Doctors . . . . | 338 |
| Variety in Diet . . . . | 338 |
| Pertaining to Insurance | 341 |
| A Question of Food . . . | 343 |
| Rattlesnakes and their Bites . . . . . . . . . | 346 |
| A Gossip with Women . . | 347 |
| Ragusa . . . . . . . . . . | 354 |
| Grand Sequences . . . . | 358 |
| The Mystery at Silver Cliff (Continued) . . . | 363 |

